# Animal culture: conservation in a changing world

**DOI:** 10.1098/rstb.2024.0127

**Published:** 2025-05-01

**Authors:** Philippa Brakes, Lucy Aplin, Emma L. Carroll, Alison L. Greggor, Andrew Whiten, Ellen C. Garland

**Affiliations:** ^1^ Centre for Ecology and Conservation, University of Exeter Faculty of Environment Science and Economy, Penryn, Cornwall, UK; ^2^ Cetacean Ecology Research Group, School of Natural Sciences, Massey University, Auckland, New Zealand; ^3^ Department of Evolutionary Biology and Environmental Studies, University of Zurich, Zürich 8050, Switzerland; ^4^ Evolution and Ecology, Australian National University Research School of Biology, Acton, ACT, Australia; ^5^ School of Biological Sciences, University of Auckland - Waipapa Taumata Rau, Auckland, New Zealand; ^6^ Conservation Science Wildlife Health, San Diego Zoo Wildlife Alliance, Escondido, CA, USA; ^7^ Centre for Social Learning and Cognitive Evolution, School of Psychology & Neuroscience, University of St Andrews, St Andrews, UK; ^8^ Sea Mammal Research Unit, School of Biology, University of St Andrews, St Andrews, UK

**Keywords:** social learning, animal culture, conservation, reintroductions/translocations, human–wildlife interactions, cultural rescue

## Abstract

Social learning and animal culture can influence conservation outcomes in significant ways. Culture is a dynamic phenomenon; socially learned behaviours can be transmitted within and/or between generations and among populations, which can facilitate resilience, or in other circumstances generate vulnerability. Culture can be a driver of evolutionary diversification, population structure and demography, shaping sociality and influencing underlying biological processes such as reproduction and survival, affecting fitness. This theme issue synthesizes the current state of knowledge on cultural variation within major vertebrate taxa, offering practical insights on how social learning can interface directly with conservation interventions. It ranges over topics that include translocations, human–wildlife interactions and adaptation to anthropogenic change. Culture is complex; integrating cultural processes into conservation is challenging. No one-size-fits-all policy can be recommended. Instead, we aim to balance current understanding of underlying processes with a diversity of practical implementations in this nascent field, exploring and supporting developing pathways towards conservation efficiencies. Key themes that emerge include conserving cultural capacity, benefits of data sharing, along with the intrinsic value of animal cultures and the role of Indigenous Peoples and local communities.

This article is part of the theme issue ‘Animal culture: conservation in a changing world’.

## 1. Introduction

Conservation aims to protect biodiversity by supporting the long-term persistence of viable, natural populations of wild species [1]. Social learning and the cultures of non-human species (hereafter ‘animal cultures’), shape the lives of many species, including such critical underlying biological processes as reproduction, migration, communication and survival [2–6]. As climate changes and ranges shift, understanding how the processes of cultural transmission shape behavioural variation will be essential for helping to predict resilience and identify vulnerability across biological systems. Genetic, ecological and demographic indicators have long been the cornerstone metrics for calculating extinction risk. However, extensive evidence for social learning or cultural transmission across a wide range of vertebrate, and possibly invertebrate, species [7,8] provides the impetus for examining how these processes influence evolutionary diversification [9], population structure [10] and demography [2,11,12]. Given the cumulative impacts on biodiversity brought about by the Anthropocene, the challenge for policy managers and conservation practitioners lies in evaluating how emergent knowledge in this field can be integrated into conservation and management strategies, in a timely and efficient manner [13–15].

In this endeavour, a broad definition of animal cultures is helpful, and we follow a conceptualization popular in the research literature on the topic, namely that ‘cultures are those group-typical behaviour patterns shared by members of a community that rely on socially learned and transmitted information’ [16]. Similarly, social learning can be most economically defined as ‘learning from others’, regardless of the information modality [17].

Despite some of the earliest scientific evidence for animal cultures being recorded in chimpanzees (*Pan troglodytes*) more than 50 years ago [18], there remains a paucity of detailed information on the phenomenon across many taxonomic groups. This has hindered necessary synthesis and the development of practical, species-specific information for conservation practitioners [19]. However, advancing methodologies [17] and evolving interest in this sphere of knowledge have resulted in animal culture becoming a rapidly emerging field. It is only when evidence is integrated with practice and policy that such knowledge can be best utilized for conservation.

Initial forays have begun to unravel the complex interface between the processes of cultural transmission and conservation [11,13,14,19], but distilling salient management advice and developing clear instructions on how precisely to integrate these fields has been more elusive [15,20]. To address this gap, this theme issue includes reviews of social learning and animal culture across vertebrate taxa, together with conservation perspectives from practitioners and managers, with the mission of providing a timely and critical contribution to this field. This introduction article first outlines some of the central questions that will help integrate insights from animal culture into conservation, then showcases the evidence for social learning across vertebrate taxa, addresses cross-cutting issues in wildlife conservation, highlights case studies that show conservation in action and finally suggests overarching themes and future directions.

## 2. Conceptual framework

**Figure 1.**
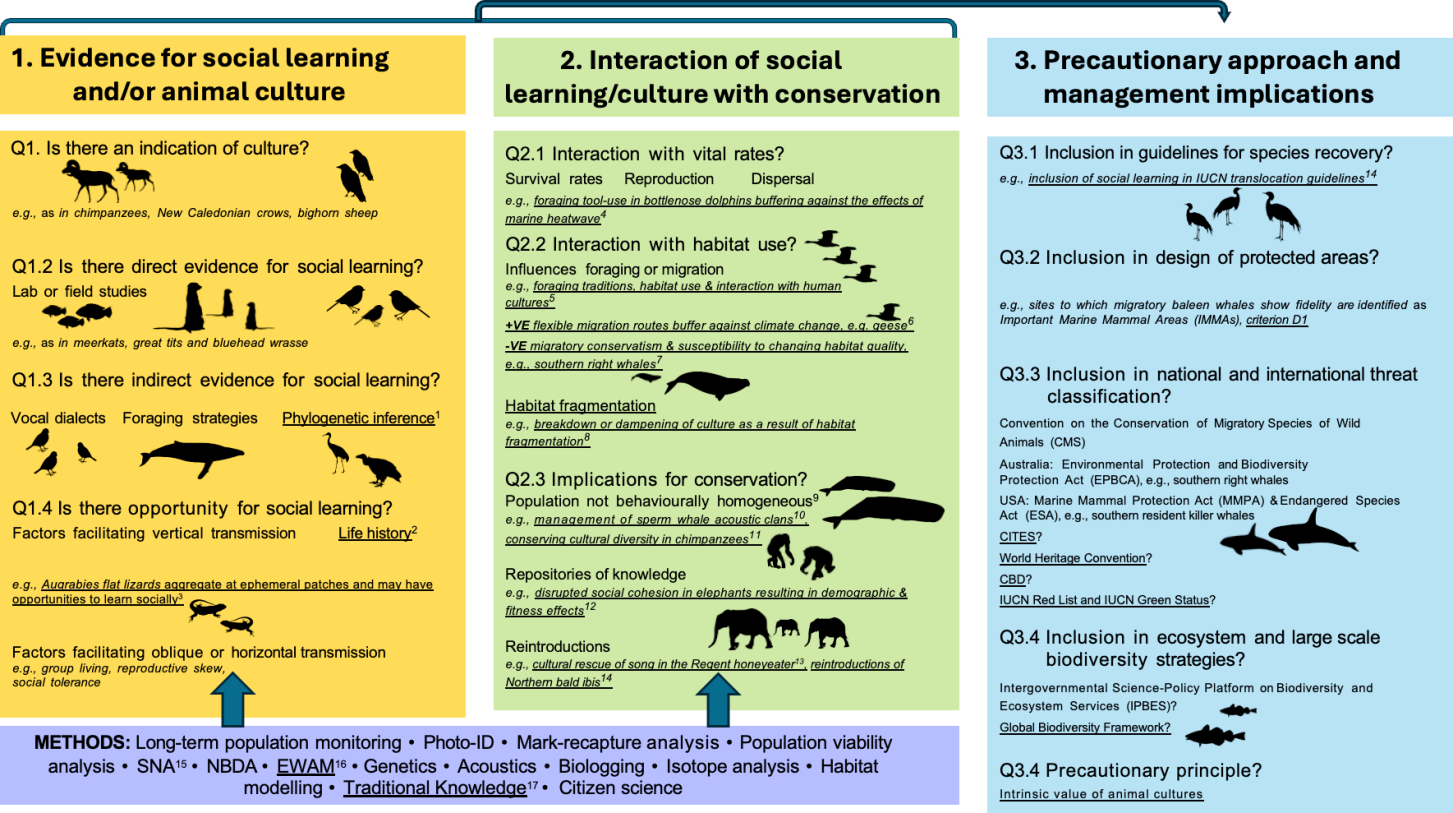
A conceptual framework for incorporating evidence and inference on social learning and animal culture into conservation policy and practice (updated after [13], additions underlined, with relevant papers in the theme issue indicated). ^1^ Aplin *et al*. [21] and Brown & Webster [22]. ^2^ Aplin *et al*. [21], Arbon *et al*. [23], Bates *et al*. [24], Brown & Webster [22], Izar *et al*. [25], Jesmer *et al*. [26] and Wilkinson *et al*. [27]. ^3^ Wilkinson *et al*. [27]. ^4^ Hersh *et al*. [28] and Brakes *et al*. [29]. ^5^ Hersh *et al*. [28] and Meaux *et al*. [30]. ^6^ Aplin *et al*. [21] and Jesmer *et al*. [26]. ^7^ Garland *et al*. [31]. ^8^ Bolcato & Aplin [32]. ^9^ Brakes *et al*. [29]. ^10^ Eguiguren *et al*. [33]. ^11^ Wessling *et al*. [34]. ^12^ Bates *et al*. [24]. ^13^ Crates *et al*. [35]. ^14^ Greggor *et al*. [36]. ^15^ Meier *et al*. [37]. ^16^ For a description of experience weighted attraction models (EWAM) and other methods, see Whiten & Rutz [17]. ^17^ Meaux *et al*. [30] and Wessling *et al*. [34]. Image credits: Chris Huh: humpback whale, killer whale, right whale sperm whale; Kent Sorgon: wrasse; T. Michael Keesey & Tony Hisgett: chimpanzee (https://creativecommons.org/licenses/by-sa/3.0/). Lauren McLean: bunting (https://creativecommons.org/licenses/by/4.0/).

To structure this issue, we employ a conceptual framework we developed earlier for integrating evidence and inference on social learning and animal culture into conservation policy and practice (figure 1, after Brakes *et al.* [13]). A first goal of the present issue is to synthesize evidence for social learning and culture across a broad range of vertebrate species, using direct evidence from lab and field studies together with more indirect evidence, such as vocal dialects that imply social learning (figure 1, panel 1). Contributors provide further guidance on methodologies appropriate to the species and ecological contexts (figure 1, methods panel); comprehensively examine the interface between social learning, animal culture and conservation (figure 1, panel 2) and finally, provide guidance on policy and management implications of these biological processes (figure 1, panel 3). We explore how advances in our understanding of animal culture is relevant to management, with contributors providing advice on augmenting existing approaches to conservation.

Noting the existing bias in the literature towards the most studied species, the issue starts with a detailed selection of contributions spanning taxa from fish to ungulates, birds and reptiles, incorporating the latest insights into social learning and culture in vertebrate species [21–28,31]. Gaps in taxonomic knowledge are identified, potential for phylogenetic inference (the plausibility of social learning occurring in related species) discussed (e.g. in fish [22] and birds [21]), and future directions indicated, including the integration of local and Indigenous Knowledge (e.g. [34]).

In addition to taxonomic insights, this theme issue includes contributions that distil cross-cutting issues on the integration of social learning and animal culture into conservation practice: for example, how social learning interfaces directly with activities such as translocations [36] or is utilized in the management of human–wildlife interactions [30]. Translocation is an umbrella term that encompasses human-mediated movement of animals for conservation purposes, and includes processes such as reintroduction, population supplementation or assisted colonization from wild or captive sources [36]. Translocations are an important conservation tool for aiding species recovery, but can be disruptive to culture, and their success can depend on the social learning of species- or population-appropriate, information or adaptive behaviour, such as the location of critical habitat or migration route [36]. Similarly, social learning can inform the management of negative human–wildlife interactions such as crop raiding [38,39], by identifying knowledgeable individuals, redirecting unwanted behaviour or seeding new behaviour [40]. The issue explores environmental predictors of behavioural diversity and cultural richness [32], and guidance is provided for researchers on methods for detecting socially transmitted knowledge, from cross-fostering field experiments to AI-assisted data mining [17]. Advice is provided on utilizing key indicators of social learning in well-studied species to provide insights and predictions concerning the behaviour of less well-studied species. For example, with only indirect evidence of social learning in elephants—but where social transmission of ecological knowledge of resource locations, such as watering holes, is considered essential for populations to thrive—the effects of disruption of putative social transmission pathways on survival and reproduction is explored [24]. Finally, contributors consider how anthropogenic disturbance and removal of animals occupying key network positions can disrupt social networks [37] and examine the complexity generated by learning biases for predicting the spread of culturally transmitted behaviour across populations [29].

Culture can be an indicator of broader ecosystem health, as when habitat conditions indirectly influence cultural richness through the size of, and tolerance for, social aggregations [32], while differences in cultural repertoire, such as foraging tactics, can create observable patterning in resource use, within or between social groups, providing a practical tool for delineating management units [20]. In a theme issue section on ‘Conservation in Action’, we explore how social learning can be manipulated to provide cultural rescue [35], and chart efforts through the Convention on the Conservation of Migratory Species of Wild Animals (CMS) (a treaty that operates under the aegis of the United Nations Environment Programmes (UNEP)), for integrating cultural processes into conservation. Proposals for concerted conservation actions spanning range states have now been approved at relevant Conferences of the Parties (COPs) for sperm whales (*Physeter macrocephalus*) [33] and chimpanzees [34]. Suggestions are also provided here for other policy fora where animal culture could provide tractable benefits, such as developing further synergies with the International Union for Conservation of Nature (IUCN) [34,36]. This is then dovetailed with efforts to maintain broader behavioural diversity [34], and the benefits of building cultural capacity across populations [13], as a source of resilience in a rapidly changing world (see [32] for discussion of urbanization).

## 3. Central questions

**Figure 2.**
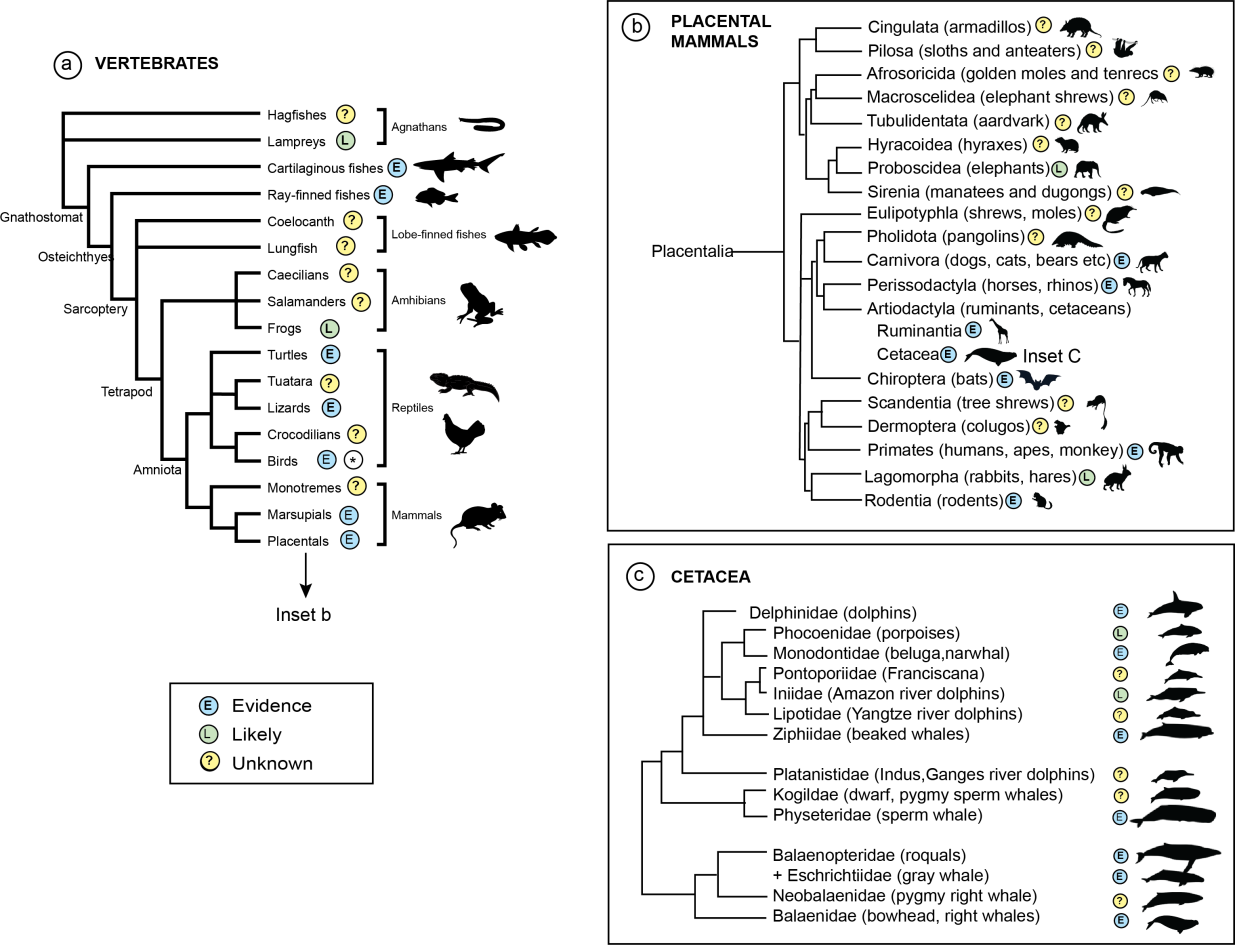
(a) Phylogenetic tree showing evidence (E) of social learning or culture across vertebrates, based on reviews within this theme issue. Wilkinson *et al*. [27] note there are at least 12 263 species of reptiles and suggests candidate behaviours that could be investigated using the lens of cultural traditions. Amphibians were not reviewed, but there is promising research in this group [41]. Brown & Webster [22], note that while there is direct evidence of social learning in bony and cartilaginous fishes, it is likely social learning may be important in many fishes. * See Aplin *et al*. [21, fig. 1] for a predictive framework for the occurrence of cultural traits across avian orders. In mammals, there is so far only very limited evidence in marsupials and no published evidence in monotremes. The spread of evidence likely reflects the distribution of research effort across taxonomic groups. (b) Phylogenetic tree of placental mammals (based on Upham *et al.* [42]) showing evidence of social learning or culture (E) and the orders where it is likely (L), or unknown (?). Mammals are among some of the better studied vertebrates, but there remain large gaps in knowledge. (c) Evidence of social learning or culture across families in the order Cetacea (E). Evidence suggests that social learning may be likely in Iniidae and Phocoenidae (L), with some of the remaining families unknown (?).

Despite the weight and diversity of emerging evidence for social learning in a vast array of wild species, understanding how culture can best be integrated into conservation actions remains challenging. The processes of social learning and cultural transmission can provide insights on '*what*' to conserve [19], often conceived as ‘the unit to conserve’ [20]. These cultural processes can also provide practical insights for '*how*' conservation can be effectively conducted [19,36]. The initial challenge has been to examine the extent to which the processes of social learning are ubiquitous in the wild. In this issue, taxonomic reviews for all major vertebrate groups reveal how widespread the processes of social learning are across the vertebrate animal kingdom (figure 2).

Cultural processes can be identified through a top-down approach, identifying patterning (e.g. different vocal dialects) between and within groups generated through cultural transmission [4,43–46] or a bottom-up approach of identifying the processes of social learning [47,48]. Fortunately, decades of research in this field have resulted in a growing methodological tool kit, with a wide range of complementary methods now available. In this issue, Whiten & Rutz distinguish 15 different approaches developed in recent decades to identify social learning and culture, each of which can now be illustrated by scores of successful examples [17]. Field experiments have arguably provided the most rigorous confirmation that behaviours of interest are transmitted by social learning in the wild, but a majority of the methods reviewed utilize only observational data—perhaps the most likely source of evidence in field settings and conservation-focused contexts.

Once the role(s) of social learning in generating spatio-temporal patterning in wild populations has been established, the next challenge is interpreting this information for conservation practitioners and policymakers. Logically, a socially learned behaviour that is adaptive and generates resilience in a population or social group is likely beneficial. For example, socially learned foraging strategies involving tool-use in a group of bottlenose dolphins (*Tursiops* spp.) buffered declining survival rates against the effects on prey of a marine heatwave [2]. However, nuance is required when applying understanding of cultural transmission to endangered or vulnerable populations and determining where to prioritize stretched resources. For some species, accounting for cultural processes or manipulating them for conservation purposes will be cost prohibitive, or less effective than other interventions, so may be ill advised. The challenge for effectively integrating insights from animal culture into conservation is determining where and when it can improve outcomes over existing methods.

To tackle these challenges, Brakes *et al*. [29], propose three central questions be addressed:

(A) How does social learning facilitate the spread of adaptive behaviour to improve the resilience of wild populations facing intra- or inter-generational environmental or ecological challenges?
(B) How do socially learned specializations, such as foraging tactics, or the social learning of less adaptive—or even maladaptive—behaviour, generate vulnerabilities in wild populations?
(C) How can the processes of social learning be harnessed to improve conservation interventions?

A key way in which these phenomena can be operationalized is for conservationists to consider whether cultural processes may be influencing mate choice, foraging or reproductive success, survival or dispersal. Aplin *et al*. and Crates *et al*. [21,35], argue that culture will most likely be of conservation concern when it creates a mismatch which hinders reproduction or decreases survival. For example, cultural mismatch between the song of reintroduced and wild populations, or reintroduced populations and the wild environment.

For conservation managers, this translates into a myriad of applied questions, such as whether advice should be to: (i) conserve cultures that generate resilience, or contain adaptive information; (ii) conserve culture in endangered species, or small isolated populations; (iii) focus on socially learned behaviour that generates vulnerability, e.g. through specialization; (iv) focus on specific content or the maintenance of social network structures so that adaptive information can be transmitted; or (v) conserve all cultures of the species concerned, irrespective of content, in the interest of biocultural diversity (after Brakes & Whitehead [49])?

To investigate these questions further, contributors to this issue explore the conditions under which cultural traits can be important for delineating a population for management purposes, particularly where cultural traits are temporally stable and where there may be differences in resource use between cultural units [26,28]. This is contrasted with instances where evidence for cultural diversity within and between social groups and populations suggests that maintaining cultural diversity may be the more salient target for conservation (e.g. in chimpanzees [34]). Examining these contrasts in different biological systems and conservation settings, contributors explore the conditions under which different approaches can genuinely assist conservation efforts. There are well documented instances where culture has been an important aspect of delineating and managing cetacean species (e.g. Southern Resident killer whales (*Orcinus ater*), beluga whales (*Delphinapterus leucas*) and southern right whales (*Eubalaena australis*) [3,20,50–52]. For example, beluga whales that summer in eastern or western Hudson Bay, Canada are listed as distinct conservation units, due to stable migratory patterns which result in consistent and different use of seasonal habitat [50]. In contrast, culture as a delineator may be less applicable for species such as chimpanzees, where a wide range of putative cultural traits vary between communities [44,53], indicating that a broader approach to conserving behavioural and cultural diversity (and likely correlates in cultural capacity and resilience) may be more appropriate [34].

Finally, beyond questions of practical application, there are more philosophical questions concerning the scale of focus for the conservation of culture. Should all cultural behaviour be conserved, irrespective of content and adaptive value in the interest of broader biocultural diversity? To what extent should we apply the precautionary principle to conserving animal cultures? Should we aim to conserve all cultures, where possible, in the interest of providing future adaptive benefit, as we do for the maintenance of genetic diversity? How does this relate to conserving the capacity for cultures to arise? Besides potential extrinsic or aesthetic value to humans, do animal cultures have intrinsic value to the species concerned [54,55], beyond their functional content [17,19]? Does animal culture have universal value as part of world heritage? Such conversations are already occurring, so setting out the major scientific underpinnings in this issue provides important grounding for these philosophical debates.

## 4. Evidence and taxonomic perspectives

While underlying biological processes shape cultural transmission, the structural features of populations and networks also shape how culture interfaces with anthropogenic threats. Here, we highlight similarities and differences between cultural transmission in vertebrate taxa emerging across eight taxonomic reviews in this theme issue. We note significant gaps in knowledge, opportunities for future research and acknowledge the differences in focus between research efforts in this field (figure 2).

Studies from birds provide extensive evidence for social learning and culture in diverse contexts from song and migration to foraging and antipredator responses [6,56–59]. For example, Crates *et al*. [35] highlight how at least 40% of the more than 10 000 bird species likely socially learn their vocalizations, often resulting in observable vocal dialects over space and, through cultural evolution, over time. Aplin *et al*. [21] evaluate emerging patterns across taxa, including cases where culture has been linked to fitness outcomes (e.g. instances where loss of vocal cultures lead to reproductive isolation [35]). Aplin *et al*. find that social learning and culture is often taxonomically conserved, but that reliance on social learning in any one domain, such as communication, is not a reliable predictor of social learning in other behavioural domains, such as migration. They note that current evidence is concentrated in a few taxonomic groups (e.g. passerines), with 21 of 37 avian orders having had little or any investigation. Given this, they suggest using a combination of phylogeny and life-history to assess whether social learning and culture is likely to influence a behaviour of concern in a species with imperfect knowledge. If the answer is potentially ‘yes’, they further recommend aiming to maintain three essential elements for culture in captive and wild populations: *capacity to re/invent*, *capacity to transmit* and *capacity to retain*.

The literature on social learning in fishes is also extensive; some of the best experimental evidence for social learning in non-humans is from experiments with fish [22]. Fish use social learning in a diverse range of contexts, and while there is a strong experimental evidence base, Brown & Webster [22] argue that cultural transmission in fishes is likely more widespread than currently understood. They note that social learning may play a particularly important role in the transmission of knowledge about habitats and argue that given the large spatial scales that many fishes inhabit, the likelihood of finding spawning or feeding grounds simply by chance is very low. They point to evidence of population-specific behaviour, suggesting either local adaptation or emergence of local culture, and indications that the timing of some migrations may be triggered by social cues [60,61]. They suggest that social learning should be the target of wider research interest in the three extant classes (Agnatha, Chondrichthyes and Osteichthyes), along with better understanding of their natural behaviours in the wild and the development of phylogenetic inference to help fill gaps. This may have practical benefits for the use of cultural transmission to better equip reintroduced fish—the primary method used to sustain threatened or endangered fish populations—with behaviours that could increase post-release survival rates. Brown & Webster also suggest that providing ‘life skills training’ [62], can significantly improve the fitness of hatchery reared fishes [63]. They urge that ‘fisheries and conservation managers should assume that many of these species do rely on social learning and culture for survival and reproduction, and manage them accordingly’, noting that dramatic population collapses of herring (*Clupea harengus*) and cod (*Gadus morhua*) were not predicted by traditional fisheries models that do not account for socially learned behaviour [22].

In contrast to many of the better studied taxonomic groups, very little is known about cultural processes in reptiles, despite their life histories including social and ecological contexts that are ‘ripe for social learning’ [27]. Nevertheless, there are tantalising preliminary insights on social learning in this group, such as evidence that chelonians, bearded dragons (*Pogona vitticeps*) and some skinks can learn from conspecifics [64–66]. Wilkinson *et al*. note that reptile embryos experience a wider range of temperatures than birds and mammals. This can impact aspects of individual phenotype relevant to social cognition and may have lasting impact on aspects of cognition and fitness relevant to their social learning and conservation. They also note the importance of chemical communication in mediating social behaviour in this group, particularly in squamates, an area that is often neglected in the study of social learning. They suggest several existing data sources, such as on sea turtle movement and bioacoustic variables that could help shed light on cultural processes in these species.

Amongst mammals, ungulates are also an often-overlooked taxonomic group. Compelling evidence indicates that social learning may facilitate ‘*green wave surfing*’—where migratory ungulates track the emergence of seasonal high-quality forage across the landscape—which is culturally transmitted across generations. Evidence following translocation of bighorn sheep (*Ovis canadensis*) and moose (*Alces americanus*), have stimulated a rethink of the role of cultural transmission in ungulate migration [67]. In this issue, Jesmer *et al*. [26] argue that the hypothesis that migration is a cultural phenomenon deserves further testing. They suggest that the vast array of migratory behaviour in ungulates may provide a form of cultural buffering to ecological fluctuations. Thus, migratory strategies may provide a useful delineator for ungulate conservation, helping to define ‘culturally significant units’ in these species.

Culture plays an important role in cetaceans’ lives, and some of the clearest indirect evidence for social learning in the wild has come from whales and dolphins. Initial forays in the Convention on the Conservation of Migratory Species of Wild Animals (CMS) into animal culture started with cetaceans. In this issue, Garland *et al*. focus on baleen whales (Mysticetes) exploring key evidence for social learning and culture and how this may interact with conservation [31]. They then suggest indicators by behavioural context to assist in identifying potential cases of social learning in more elusive species. For example, in the context of foraging behaviours, humpback whales (*Megaptera novaeangliae*) [47] and Bryde’s whales (*Balaenoptera edeni* spp.) [68] have highly plastic but socially learned foraging tactics that allow for prey switching. This illustrates the potentially complex intersection between social learning and conservation [69]. Foraging plasticity and behavioural change within a population can be seen as strong potential indicators of social learning along with consistent differences between populations. In the context of migratory traditions, right whales (*Eubalaena* sp.), for example, show fidelity to migratory terminals that is likely socially learned, as well as variation when individuals move to other areas [3,70–72]. Cultural knowledge of migratory habitats and shifting habitat use in response to climate change illustrates a mix of behavioural conservatism and plasticity. Finally, shared, rapidly changing song dialects are strong indicators of social learning in the context of vocal communication. Both humpback [4] and bowhead whales (*Balaena mysticetus*) [73] display vocal plasticity and rapid change that cannot be explained without social learning. The case studies highlighted in Garland *et al*. [31] illustrate the importance of social learning in foraging, migration and vocal behaviour for baleen whales. The authors additionally highlight that social learning in baleen whales may also be supported by other traits, such as memory (e.g. blue whale, *Balaenoptera musculus* spp. [74]; humpback [75]) and communication (e.g. feeding call facilitating coordination in humpbacks).

Whilst a handful of the odontocetes (or toothed whales and dolphins) are among some of the best-studied species in this field [76], new insights are still emerging. For example, observational evidence of Baird’s beaked whales (*Berardius bairdii*) indicates that knowledge about habitat use is socially transmitted in this beaked whale species [77]. Given the breadth of evidence across multiple behaviour domains in odontocetes [76], and the potential for culturally transmitted foraging behaviours to profoundly impact population dynamics, Hersh *et al*. [28], focus on foraging tactics and their potential significance to species ecology and conservation, as a practical means of operationalizing insights on socially learned behaviour. They provide a global inventory of foraging tactics in odontocetes, evaluate the role of social learning, consider the implications for conservation and suggest potential safeguards. Their meta-analysis identifies 55 putative cultural foraging tactics, with 50.9% providing quantitative support for social learning and 49.1% with qualitative support. The vast majority of those with quantitative support (82.1%), indicated a correlation between social structure and foraging tactic use and eight of these cases ‘had enough evidence to likely indicate discreteness and/or evolutionary significance of potential conservation units’ [28], with evidence of vertical learning being the most common. They highlight the challenges, particularly for the more enigmatic, or harder to study, species and consider how the discreteness and evolutionary significance of some cultural foraging strategies may help delineate populations for conservation effort [20]. They also note that some ‘anthropo-dependent’ [78] foraging tactics, such as depredation from long-lines or human provisioning, that may seem advantageous in the short-term, may not necessarily be so in the longer-term if they result in reduced survival or reproduction [12].

As Izar *et al*. [25] illustrate, some of the most extensive and compelling evidence for animal culture has accrued across the primate order during the last seven decades. If these discoveries can be harnessed to facilitate conservation, this is now urgent for the order. Recent reports conclude that as many as 60% of the roughly 500 species of primate are now ‘threatened with extinction’ [79] and population decline is documented for over 75%. Izar *et al*. note that, remarkably, the rich evidence for primate culture has been derived from only around 3% of these primate species to date. Adopting the precautionary principle, they urge that given the existing evidence that social learning and culture pervade primates’ lives, this should be a strong assumption for most primate species where it concerns conservation efforts [25,80]. Many species have been heavily impacted by anthropogenic changes, which Izar *et al*. link with cultural processes in both negative and more positive ways: black howler monkeys (*Alouatta pigra*), for example, have shown much diminished behavioural repertoires where forest refuges have been eroded [81]; whereas interfacing with tourism has led to such developments as long-tailed macaques (*Macaca fascicularis*) bartering stolen sunglasses and the like for nutritional payoffs, with a human/non-human cultural arms-race of opposing tactics [82]. Izar *et al*. develop a series of recommendations for the furtherance of linkages between the science of primate culture and conservation efforts.

Reviewing mammalian groups not covered elsewhere and reflecting on process-based issues beyond mammals, Arbon *et al*. [23] highlight the degree of flexibility in social learning across taxa, both in terms of when social information is used and who individuals learn from. They note that there is often a critical period for learning early in life; that social learning has even been shown to begin *in utero* in rats (*Rattus* sp.) and that young meerkats (*Suricata suricatta*) and smooth-coated otters (*Lutrogale perspicillata*) are more likely to socially learn new foraging techniques than adults [23]. Similarly, from whom individuals learn may also depend on environmental stressors, e.g. experimental evidence in zebra finches (*Taeniopygia castanotis*) shows strategy switching under stress, between learning from parents, to learning from non-parent demonstrators [83]. Data on ontogeny can be applied to practical applications, such as removing access to learning opportunities during critical periods for vertical transmission, to reduce undesirable human–wildlife interactions. For example, in managing brown bears (*Ursus arctos*), closing picnic areas when informed females with dependent offspring are in the vicinity may reduce vertical learning opportunities and therefore reduce the probability of future conflicts [84,85]. They also emphasize the need to ensure opportunities for social learning in pre-release of captive reared mammals, with post-release monitoring to assess the knowledge and fitness of reintroduced individuals. They recommend forums such as ‘Conservation Evidence’ [86], for intervention outcomes to be shared with conservation practitioners. They emphasize that the specific factors that drive differences in social learning strategies and flexibility within and across taxa are important targets for fundamental and applied research in this field.

In contrast to other taxonomic reviews, evidence on elephants presented by Bates *et al*. [24] acknowledges the conundrum that while cultural knowledge is widely assumed to be relevant for all three species of elephant, direct evidence of social learning and culture is scarce. To address this gap, Bates *et al*. take a novel, reverse engineering approach by examining the consequences of social disruption, through natural or anthropogenic causes. Their meta-analysis of 95 peer-reviewed articles shows that for the most severely disrupted populations there are consequences for social cohesion that may result in demographic and fitness effects, such as reduced calf survival, or impacted ability to respond appropriately to threats and predators. This indicates that severe social disruption can inhibit or break potential pathways of information transmission. Their analysis—and others in this issue [37]—provide further process-based insights on the effects of social disruption and offer food for thought on how thresholds for the Allee effect [87], where a population can tip into rapid decline at low population densities, may be influenced through the disruption of information pathways [29,88].

## 5. Cross-cutting issues in wildlife conservation

It is essential to develop a common language in this field so that scientific insights can realistically be integrated in a practical and timely manner. Synthesizing this body of evidence, the grand challenge from a management perspective is then distilling this into cogent management advice that can be implemented in practice (a common problem in conservation [89]). One approach to this has been to take a process-based approach that examines underlying, ultimate causation of the patterning generated by social learning and culture, to determine themes that might be applicable across different taxonomic groups [29,32,37]. Another approach is to examine the role of social learning in behaviour important for specific conservation activities such as translocations [36], or for particular types of cultures (e.g. vocal cultures [35]).

Bolcato & Aplin [32] ask how the observed variation in the diversity, complexity and richness of animal culture is influenced by variation in the environment. They find striking commonalities across species and behaviours, for example finding that habitat heterogeneity and variability tends to support greater cultural richness in taxa and behaviours as different as tool use in chimpanzees [53] and song in passerine birds [90]. The authors use these findings to create a predictive framework for cultural change in the Anthropocene, focusing on the major threatening process of habitat degradation, habitat fragmentation and urbanization. They find that habitat degradation and fragmentation in particular are predicted to have both direct negative impacts on culture and indirect negative impacts via changes in sociality and behaviour, which together will tend to erode cultural richness and complexity. These changes, in turn, may exacerbate declines by reducing the capacity of animal populations to adapt to changing conditions. They emphasize the importance of maintaining heterogenous, productive and connected habitats to support cultural resilience in animal populations.

Evaluating the human cultural dimension of conservation, particularly as it relates to human–wildlife interactions (HWI), is also essential, because conservation requires human actions at multiple scales. Exploring the idea that conservation outcomes will often be influenced by behaviour—either human or non-human—Meaux *et al*. [30] examine the language used to analyse human–animal interactions and how theory and practice can be used to negotiate some of these complex interactions. Noting that these behavioural processes can operate at different conservation scales, they provide concrete recommendations for integrating understanding of culture and social learning during HWI into conservation actions. These include the need for pre- and post-implementation engagement with all stakeholders, the need to develop a central repository on novel behaviours associated with negative HWI outcomes, and the need to develop methods to evaluate the reciprocity between human and non-human behaviours. They emphasize the role of communication and education about animal cultures to improve local attitudes towards animal conservation and HWI. They also note the need to develop methods to analyse how the transmission of information across animal social networks can be modified by humans to improve predictions about the outcomes of HWI and provide opportunities for early intervention (at the interaction phase), to prevent these interactions evolving into conflicts.

Some conservation subdisciplines have started implementing practices that retain, foster and encourage socially learned behaviours, without needing to invoke culture explicitly. For example, in species translocations, wherein animals are disjointed from source populations, either from captivity or the wild, and moved into areas where other cultures exist, or to areas where no conspecifics are present, awareness of the fitness impact of a potential behavioural mismatch is growing [35,91]. Practices such as pre-release training, fostering coherent social groups and tracking integration into wild populations are not uncommon. However, the explicit study, recognition and targeting of cultural processes remains rare. Across the diverse types of translocations, the consequences of ignoring socially learned behaviour can be disastrous, and result in the failure of translocation efforts, as when translocated animals do not have access to knowledge about critical habitat or resources, or where ‘conflict individuals’ are relocated with low success rate [35]. Despite these major pitfalls, direct action to preserve cultural behaviour pre-release or reinstate it post-release can vary in difficulty depending on the specifics of the behaviour and time lag from learning to its expression. The challenge lies in determining which socially learned behaviours are key, and when to intervene [21]. Greggor *et al*. propose a ‘traffic light’ framework for decision making to help guide interventions, and also offer specific recommendations for where existing international guidelines can be updated to better reflect the emerging evidence on the utility of explicit cultural intervention [36].

Another valuable addition to this cross-cutting section by Whiten & Rutz [17] explores the growing methodological tool kit developed from decades of research in this field. They distinguish an enormous diversity of approaches to identifying social learning and culture, illustrated by as many as 80 concrete examples of successful studies across all major vertebrate taxa [17]. Those researching the most appropriate methods for their own scientific and conservation projects should find much inspiration here. In addition to field experiments, the majority of these studies involve a constantly evolving and ingenious range of ways to interrogate observational data for evidence of cultural phenomena. Methods include assessing whether innovation spreads over known social networks, comparing the repertoires of neighbouring groups (ruling out environmental and genetic explanations) and measuring the relative impacts of opportunities for social as opposed to individual learning. For example, a gold standard technique in this field is the use of network-based diffusion analysis (NBDA) to interrogate observational data by establishing whether the transmission of a novel behaviour maps onto social network structure [47].

Two final contributions consider culture and conservation from the perspective of underlying processes that drive some of the outcomes and culturally mediated granularity that can be detected in wild populations. First, Meier *et al*. [37] explore the spread of innovations across social networks. Second, Brakes *et al*. [29] examine the conditions under which social learning can scale up to have population level effects, when changes in reproduction, survival or dispersal can influence population parameters such as growth rate. These contributions consider both the shorter and the more medium-term perspective on the interface between animal culture and conservation, examining the role of key individuals and learning biases.

Meier *et al*. [37] use long-term studies on dolphins, elephants and baboons to explore how network indicators can identify resilience to anthropogenic threats generated through cultural transmission [37]. By simulating anthropogenic removals from within social networks they provide insights into both the role of key, well-connected individuals and the role of innovation in social learning. Supporting the work of others [92], they show how removing key network positions, rather than age-specific or random individuals, negatively affects social networks. This work also shows that high levels of innovation within a group can generate resilience by mitigating the fitness cost associated with individual removals and demonstrates the need for identifying and protecting key individuals to mitigate anthropogenic disturbance.

While there is a strong impetus to develop pathways from the theory of cultural transmission to conserving animal culture in practice (see §6), there is a concurrent need to develop deeper understanding of the underlying processes to make better predictions and identify potential scenarios where social learning could be exacerbating a conservation crisis. Brakes *et al*. [29] note that in concert with feedback from empirical research and efforts to improve conservation outcomes in practice, there remains a need to resolve some fundamental principles on the many ways in which social learning and animal culture can interface with population behaviour and conservation activities. By combining theoretical research with empirical studies and practical conservation efforts, we can develop more comprehensive and effective strategies to protect biodiversity and ensure sustainability, whilst balancing this with taking urgent action for the many imperilled species and populations.

## 6. Conservation in action

An emergent challenge in this field is the translation of data on social learning and culture into application [15]. Integrating cultural considerations into conservation practice will require more than just the production of relevant data. Insights should be communicated and packaged appropriately so that they can be shared beyond peer review articles, to reach conservation practitioners for their feedback. For instance, the IUCN’s translocation guidelines offer interdisciplinary best-practice advice, used by practitioners worldwide. Greggor *et al*. [36] suggest ways in which equivalent policy forums for expanding best practice will vary by type of conservation intervention or taxonomic group. Three ground-breaking case studies highlighted in this issue demonstrate some of the complexities involved and the need for tailored approaches: work conducted under CMS for sperm whales in the eastern tropical Pacific [33], under the IUCN and CMS for western chimpanzees [34], and captive-breeding programmes for critically endangered regent honeyeaters, *Anthochaera phrygia* [35]. There is not a ‘one-size-fits-all’ solution in this field. There must be careful evaluation of a range of situation-dependent variables. Life history, behavioural domain, cultural variant, social learning biases and transmission pathway are all important biological factors [29]. Conservation outcomes being realized will depend on integration with existing conservation efforts, the human cultural element and the multi-level political landscape (from local to international).

Sperm whales are separated into groups (clans) in the eastern tropical Pacific (ETP) based on their vocal cultures, with evidence of variation in foraging tactics between clans raising questions about different clans' ability to respond to threats such as climate change [33]. Reviewing progress on a CMS Concerted Action for sperm whales in the eastern tropical Pacific, Eguiguren *et al*. [33] describe setting up a collaborative research network with sperm whale researchers across the region: from Mexico, Costa Rica, Panama, Colombia, Ecuador, Peru and Chile [33]. This is the essential first step to allow conservation initiatives to operate over the large ranges that sperm whales traverse. This exemplary network, in a region with limited research and conservation resources, collaborates to share data and methodologies, including for detecting social learning within and between clans. The participants connect, share knowledge and help build capacity within the region to explore the many questions relating to the structure of the acoustic sperm whale clans in this region. The objective of this work is to evaluate the potential threats at the clan level and to establish whether sperm whale cultural clans in this region—identified by their unique, socially learned, acoustic codas—should be managed as culturally significant units. Eguiguren *et al*. [33] outline some of the ongoing challenges of incorporating culture dimensions into the conservation of sperm whales. They highlight the potential benefits of new technologies to help with data collection and analysis but also emphasize the need to strengthen transnational collaboration for the conservation of this species and other highly mobile oceanic species whose behaviours are shaped by social learning (see [22,28,31]). They also note the need to establish equitable and inclusive collaborations between ETP nations and high-income countries to facilitate local-capacity building.

Examining further CMS Concerted Actions on culture in chimpanzees, Wessling *et al*. [34] review approaches to date under both CMS and the IUCN Section on Great Apes. Given the wide diversity of putative cultural behaviours recorded in chimpanzees [53], the pervading role of culture in chimpanzee behaviour and the practical challenges associated with chimpanzee conservation, it is suggested that, supporting cultural diversity, and thence the overall resilience of the species, is an optimal target for chimpanzees. Concerns about the potential prioritization dilemmas thought to be inherent in an initial, exploratory Concerted Action focused on the specific endangered culture of nut-cracking among critically endangered Western chimpanzees, led to this initiative being superseded by a more ambitious Concerted Action to support cultural diversity across the species’ range, approved at a CMS Conference of the Parties in 2024 and now being pursued by a collaboration between members of CMS and IUCN.

The budding concept of cultural rescue is also gaining traction, as researchers and practitioners collaborate to revitalize avian song cultures, which can be lost through erosion/fragmentation, divergence and convergence. Crates *et al*. [35] use recent attempts to conserve and recover song culture in critically endangered regent honeyeaters as a case study to highlight the loss of avian vocal cultures more broadly. They discuss the drivers and consequences of the erosion of avian vocal cultures, with examples ranging from habitat fragmentation leading to fewer tutors in Albert’s lyrebirds (*Menura alberti*), resulting in song simplification [93]; to habitat loss driving niche overlap in three species of Hawaiian treecreepers, resulting in song convergence as individuals rely on heterospecific tutors [94]. In regent honeyeaters, drivers of song culture loss have been twofold. In the wild, low population densities has led to juveniles having fewer opportunities to learn from older males, leading to song simplification and the incorporation of heterospecific songs [5]. In captive populations, breeding programmes which aimed to increase fecundity by separating young from adults, led to a highly simplified ‘babbling’ song. In all cases, males with songs that differ from cultural norms have low reproductive success, increasing wild declines and negating reintroduction efforts. Crates *et al*. [35] provide a detailed account of recent successes in retraining captive populations to wild-type song cultures, with future hopes to perform a ‘cultural rescue’ of wild vocal cultures via reintroductions. This work demonstrates the importance of integrating understanding of culture into captive breeding programmes and showcases how doing so has the potential to dramatically increase the success of reintroduction efforts [21,35].

## 7. Themes and future directions

This theme issue offers a horizon scan of the current state of knowledge and evidence in this field [21–28,31], noting technical advances and opportunities [17]. Successful implementation also often requires consideration of the human factor [30], as well as a deeper understanding of the underlying processes, such as social learning biases [29], social networks [37], environmental predictors [32] including phenological mismatches [23,26] and precision concerning the exact targets for conservation action [23,29,33,34,37]. Finally, practical advice on implementation is beginning to emerge from this knowledge for conservation actions such as reintroductions and translocations [35,36].

A key theme is the question of how to support adaptive cultural variants that confer fitness benefits, as these can provide a compelling target for conservation intervention [29,35]. It may be possible to achieve this by maintaining existing cultural diversity [34], or, more broadly, by working to conserve cultural capacity across populations [13]. Cultural capacity can be maintained by supporting the conditions for adaptive variants to arise [32], or by supporting the conditions for population-wide cultural transmission of shared communication systems such as bird song [21] or whale song and codas [28,31]. To address this, we need to identify principles that can be applied across multiple systems, with combined insights from theoretical and empirical ecologists [29]. Understanding the population consequences of social learning is also important, as whilst stable intergenerational cultures may provide insights for delineating some populations [20], transient or temporary suboptimal, culturally transmitted behaviour can also impact survival or reproduction (and possibly dispersal) and may require attention, as in crop raiding [38,39] and depredation [95].

**Figure 3.**
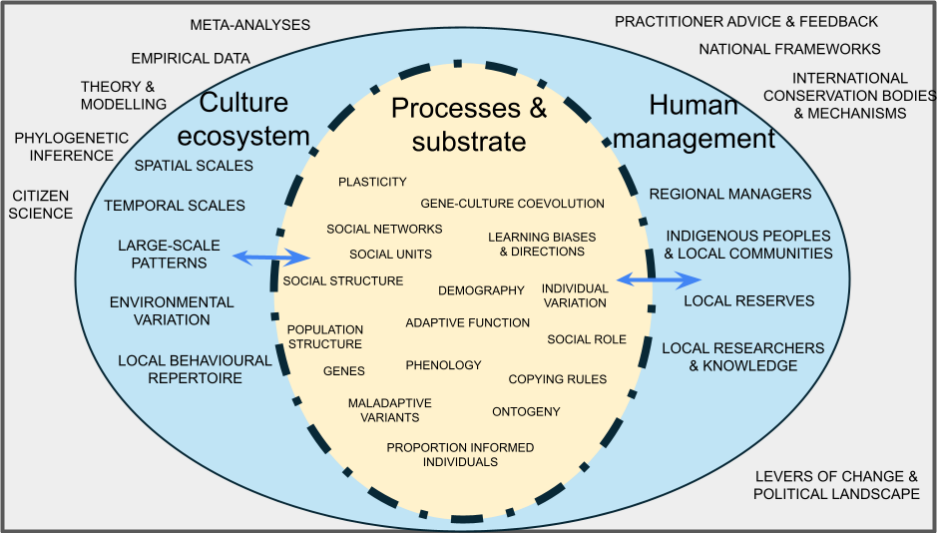
Eye of cultural complexity. The multiple dimensions of culture and conservation. Central layer: processes and substrate layer, showing that social learning and cultural processes act on individuals, groups, populations and social structures, noting the potential for bidirectional feedback between layers. Outer layer: human focused and broader scale processes (both biotic and abiotic) that influence how culture may manifest at multiple scales. Outside: (left) information sources that inform understanding of cultural variation across ecosystem; (right) opportunities for conservation implementation. This list is not exhaustive but intended to provide perspective on the number of interacting processes and layers, which can also include bidirectional feedback.

There is good evidence that in some cetacean species, where cultural traits, such as foraging tactics, remain temporally stable, delineation between cultural units can be a useful management tool [20,28]. However, there are many instances where identifying specific targets for action may be challenging, or even inappropriate, due to the granularity and complexity with which different cultural traits manifest or can be measured across the population, or field constraints and implementation factors [34]. Generally, we suggest maintaining cultural diversity through specific activities associated with supporting the capacity for cultural transmission to emerge in these populations. In the gambit of potential innovations that may arise, we define maintaining ‘cultural capacity’ as supporting all the abiotic and biotic features required for innovation and social learning to be maintained within a population, to increase the probability of locally adaptive variants arising and being transmitted. To understand how to conserve cultural capacity within a population, one must first consider the underlying processes and the multiple dimensions of social learning and cultural transmission, specifically as they relate to the domains of conservation. Figure 3 highlights some of the complex, interconnected dimensions of animal cultural processes and how these relate to multiscale conservation activities.

The central objective for maintaining cultural capacity is to support both existing adaptive cultural variants and the conditions necessary for novel adaptive variants to be generated and propagated. Maintaining cultural capacity differs from conserving behavioural diversity—a similarly laudable goal—by specifically identifying and conserving features associated with the maintenance and transmission of adaptive variants. Whilst this will differ on a case-by-case basis, in the broadest sense, this equates to the maintenance of healthy ecosystems and the optimal environmental conditions for transmission. In other words, maintenance of all the biotic and abiotic features required to support both innovation and cultural transmission. This ecological integrity includes features such as diverse habitat and stable prey-base, through to ensuring that the materials for any tool-use are available, supporting connectivity and reducing fragmentation to maintain cultural transmission pathways, strong network connections and stable population structure. Maintaining the social integrity of populations and the pathways for social learning can help facilitate real-time responses to intra-generational environmental and ecological challenges. Possibly the most important element of these is maintaining population density beyond basic ‘effective population size’, with consideration for elements such as age structure, which can influence adaptive cultural transmission between naive and informed individuals and cohorts [29].

In practical terms, this can include maintaining connectivity between social groups and populations, such as corridors between habitats and exchanges between island populations. Managers should also be aware how Allee effects [88,96], and other population thresholds that can bring about precipitous decline, could be compounded by lack of connectivity, low population abundance and any barriers to transmission, such as anthropogenic noise [97]. This practical suggestion of supporting cultural capacity, to help provide the necessary features for adaptive cultural variants to arise [19], also supports philosophical arguments for recognising the intrinsic value of animal culture, irrespective of its functional value.

Remaining questions for future work in this field include: *what* precisely should we aim to conserve and *why*? The question of whether to protect specific cultural variants for their functional value to resilience and/or utility to conservation efforts can be contrasted with an objective of conserving cultural diversity and cultural capacity more broadly, due to culture in other species having intrinsic value. Here we take the ‘intrinsic value’ of animal culture not just to mean the value of animal culture to the humans who interact with these cultural species and groups, either through functional or aesthetic value, but also in terms of the intrinsic value of these specific cultural ways of behaving to the animals themselves. While inherently difficult to evaluate the intrinsic value of animal culture, it can be part of wider considerations. We caution against simply assessing the merit of cultural diversity in other species for its mechanistic value or utility to conservation alone, as we do, to a large extent, with genetic diversity. What are the implications of self-medicating in primates [98], or evidence for tool-use in fishes [99,100]? What is the intrinsic value of whale or bird song or a specific behaviour such as adornment with grass [101] or tail-walking in a wild bottlenose dolphin population, that may act as an ethnic marker [102]? How do such value judgements differ across human cultures? Should cultural behavioural variation be considered under the wider umbrella of natural heritage conservation [22,54]. How might this be achieved in practice?

Whilst many questions remain, it is undoubtedly the case that animal cultures have been in existence much longer than we have had the tools to document them. Earliest evidence on animal cultures came from studies of chimpanzees from more than 50 years ago [18], while fossil evidence indicates the use of stone tools in chimpanzees dating back around 4300 years [103]. Although modern science may have been slow to develop tools to recognize cultural transmission in species beyond our own, some Indigenous Peoples and local communities have long understood the transmission of knowledge between ourselves and other species, through intergenerational mutualism between a range of species from birds to killer whales [104–107]. Integrating different streams of knowledge with this depth of understanding on people and place will be an essential part of this field moving forward. Using appropriate frameworks for engagement will enable a more comprehensive approach to the goal of conserving both the intrinsic and the functional value of animal cultural diversity.

Finally, we provide some general recommendations to help streamline and safeguard the integrity of this developing field:

— A key overarching goal is to maintain cultural capacity within and between populations, to ensure adaptive cultural variants can be invented, transmitted and maintained in response to novel stressors.
— Where appropriate, explore fitness consequences of cultural variants.
— Consider when cultural mismatches between individuals and social or physical environments may lead to reduced fitness, as for cultural conformity in bird song.
— Conserve cultural behaviours that may change in content but are functionally essential, e.g. bird and whale song.
— Develop tools to support decision making [108,109], such as the traffic light system proposed by Greggor *et al*., for translocations [36].
— Support multi-decadal longitudinal observational research, but where this is not practical, develop tools for plausible phylogenetic inference to identify instances where social learning provides resilience or vulnerability and offer guidance on where to focus limited resources
— Be clear about what managers need to do and why. Ensure feedback from conservation practitioners and Indigenous Peoples and Local Communities (IPLCs).
— Share information about suspected impacts of cultural processes on conservation interventions in peer reviewed papers or open access forums.
— During HWI, ensure pre- and post-implementation engagement with all stakeholders and develop a central repository on novel behaviours with negative HWI outcomes.
— Identify levers of change by exploring the interface between human and animal cultures and the realities of the political landscape.
— Strengthen transnational collaboration, share methodologies to build capacity, particularly with early career researchers in the Global South.
— Continue to explore how new evidence in this field can inform activities across national frameworks and through international conservation bodies such as CMS and IUCN.

## 8. Concluding remarks

Pioneers in this field, Whitehead & Rendell noted that ‘Culture complicates conservation and it complicates it in all kinds of complicated ways’ [76]. Culture is indeed complex and how culture interfaces with conservation is multifaceted. In some areas, advice can be distilled (e.g. [30,36]), to increase conservation efficiencies or help define the ‘unit to conserve’. However, there is still a great deal to learn about the interplay between social learning and social structure, social networks, demography, connectivity and genes. This field is challenging, but when thoughtfully integrated, significant benefits can be accrued; complementary to, and augmenting, existing conservation efforts.

As we delve deeper into the diversity and heterogeneity created by social learning and culture, and how understanding in this field can augment existing conservation efforts, the layers of complexity unfold (figure 3). This theme issue showcases evidence for this complexity across major vertebrate taxonomic groups, highlights similarities and differences across different systems, as well as highlighting the need for fundamental principles in this field. By exploring in detail some of the many implications for conservation, we highlight central questions that can provide a foundation for these efforts moving forward.

Culture generates behavioural diversity in wild populations that can result in vulnerability or generate resilience. Efforts to integrate culture into conservation can be approached from various perspectives: examining the underlying processes, observation of the patterning created by cultural processes, or though examining the role of human behaviour. In some instances, culture can be useful for delineating ‘units to conserve’; it can also improve practical conservation interventions such as translocations or during HWI; in many cases there is a strong argument for conserving cultural diversity by supporting cultural capacity within and between populations to ameliorate the heterogeneous impacts of climate change. In synthesising the many contributions to this theme issue, we offer some guidance on how conserving cultural capacity within and between populations might be achieved. We note that efforts should focus on ensuring efficacy and efficiency by harnessing the processes of social learning to offer genuine opportunities for practical gains, thus expanding the scope and reach of conservation through animal culture.

Finally, we argue that animal cultures may have intrinsic value, not just to those human cultures that observe and interact with them, but perhaps for the animals themselves. This generates a new paradigm for exploration, in which the preservation of animal cultures, for their own sake, is not a zero-sum game based on conservation utility, but instead serves to broaden the breadth and scope of our engagement with and understanding of the natural world.

## Data Availability

This article has no additional data.
